# Clinical Variables Associated with PSA Response to Lutetium-177-PSMA ([177Lu]-PSMA-617) Radionuclide Treatment in Men with Metastatic Castration-Resistant Prostate Cancer

**DOI:** 10.3390/cancers12051078

**Published:** 2020-04-26

**Authors:** Moran Gadot, Tima Davidson, Margalit Aharon, Eshetu G. Atenafu, Avraham Malki, Meital Levartovsky, Akram Saad, Liran Domachevsky, Raanan Berger, Raya Leibowitz

**Affiliations:** 1 Oncology Institute, Sheba Medical Center, Tel-Hashomer 52621, Israel; 2 Department of Nuclear Medicine, Sheba Medical Center, Tel-Hashomer 52621, Israel; 3 Sackler Faculty of Medicine, Tel-Aviv University, Tel-Aviv 6997801, Israel; 4 Department of Biostatistics, Princess Margaret Cancer Centre, Toronto, ON M5G 2M9, Canada

**Keywords:** Lutetium, theranostics, prostate cancer, mCRPC

## Abstract

Lutetium-177-PSMA ([177Lu]-PSMA-617), a radiolabeled small molecule, binds with high affinity to prostate-specific membrane antigen (PSMA), enabling targeted radiation therapy to metastatic prostate lesions. Our objective was to retrospectively analyze the activity of [177Lu]-PSMA-617 given off-trial to men with metastatic castration resistant prostate cancer (mCRPC) and identify clinical factors associated with PSA response. Electronic medical records of all men treated with [177Lu]-PSMA-617 were reviewed and analyzed. Overall survival was calculated using the Kaplan–Meier method. The association between potential variables and PSA response was analyzed by univariate analysis, using either logistic regression or χ^2^/Fisher’s exact test. Multivariable analysis was carried out using logistic regression on all categorical variables with a *P*-value of <0.1 on univariate analysis. Variables found to be statistically significant were then used to define a categorical score. A total of 52 patients received at least one cycle of [177Lu]-PSMA-617. Clinical benefit was observed in 28 patients (52%). PSA decline ≥20% and ≥50% was observed in 26 (50%) and 18 patients (35%), respectively. Achievement of any PSA decline at first measurement was significantly associated with survival. There was a negative association between the number of previous chemotherapy lines and PSA decline above 20%. Univariate analysis followed by multivariable analysis showed that older age and higher hemoglobin were significantly associated with a PSA decline >20%. A score combining these two parameters was significantly associated with PSA response. In summary, [177Lu]-PSMA-617 is active in the ‘real-life’ setting of heavily pretreated men with mCRPC.

## 1. Introduction

Metastatic castration resistant prostate cancer (mCRPC), the last phase of the prostate cancer continuum, continues to be a fatal disease [1]. In the first half of the 2010s, there was unprecedented progress in the treatment of mCRPC, with the addition of four new survival-prolonging drugs to the armamentarium of treatment options—abiraterone acetate, cabazitaxel, enzalutamide, and radium-223 (reviewed in [2]). In marked contrast, in the second half of the 2010s, there were no positive phase III randomized controlled trials, and progress seemed to have reached somewhat of a halt.

Against this background, the last decade has seen the invention and development of radiopharmaceuticals that target specific membrane-bound proteins in a few types of cancer, a therapeutic approach designated ‘theranostics’. Prostate-specific membrane antigen (PSMA), a transmembrane protein highly overexpressed in cells of prostatic origin, is the target of several such radiopharmaceuticals, which differ in their biophysical properties. Of these, Lutetium-177-PSMA ([177Lu]-PSMA-617) was found to be most promising and has emerged as the leading compound to date (reviewed briefly in [3]). [177Lu]-PSMA-617 binds with high affinity to PSMA and is a mixed beta-gamma emitter that causes cellular damage to PSMA-expressing prostate cancers cells in a relatively specific manner, thus sparing adjacent normal organs and tissues [4].

For several reasons, the development of [177Lu]-PSMA-617 as a therapeutic modality did not follow the usual drug-development path of phase I–III clinical trials, and its administration as a palliative last (or almost-last) treatment line preceded the execution of formal randomized controlled trials. The most advanced published clinical trial until now is a phase II single-arm trial 5, showing very promising palliative results. Based on accumulating global clinical experience and in a similar manner to other sites across the world, [177Lu]-PSMA-617 has been offered through an off-trial, self-paid mechanism at our institution since 2017. This retrospective analysis describes our clinical experience with this compound and the clinical and laboratory parameters associated with biochemical response to treatment.

## 2. Patients and Methods

### 2.1. Patients

[177Lu]-PSMA-617 was offered, on an off-trial, self-funded basis, to men with mCRPC who (all but two) had exhausted all lines of therapy or were deemed unfit to receive any additional approved lines of therapy. Men were eligible if they had a PSMA-positive scan up to six months prior to treatment.

### 2.2. [177Lu]-PSMA-617 Treatment

A DKFZ-PSMA-617 precursor (ABX, Germany) was radiolabeled with no-carrier-added-Lutetium-177 chloride ([177Lu]-Cl3; Isotopia Molecular Imaging Ltd.) in Isotopia’s Radiopharmacy according to the manufacturer’s instructions. The precursor (200 μg) was diluted with 700 μL of 0.4 M sodium acetate buffer pH 5.0 (Merck, Germany), containing 4 mg of gentisic acid, and added to 10 GBq of [177Lu]-Cl3 in 0.04 mol/L HCl. The solution was heated to 100–105 °C for 45 min with intermittent gentle agitation. After the cooling down of the reaction, the volume was adjusted to 5 mL using 0.9% sterile saline. Radiochemical purity was established by high performance liquid chromatography. [177Lu]-PSMA-617 was administered by slow intravenous injection over 2–10 min. Patients were encouraged to be well hydrated by consuming oral fluids on the day of the [177Lu]-PSMA-617 administration. After the first cycle, quantitative single photon emission CT/CT (qSPECT/CT) scans from the vertex to the thighs were acquired at 4 and 24 h, enabling quantitation of [177Lu]-PSMA-617 retention within tumor and normal tissues. For subsequent cycles, a single-timepoint 24 h qSPECT/CT was acquired.

### 2.3. Clinical Assessment

PSA, blood count, and additional blood tests were documented prior to the first treatment (‘baseline’) and throughout the follow-up. Patients returned to the clinic for safety assessment within 4 weeks after the first treatment cycle, and then according to physician’s discretion. Complete blood tests including PSA were performed for each preplanned clinic visit and between visits upon need. The ‘first PSA’ was defined as the first PSA measurement following the start of treatment. ‘PSA nadir’ was defined as the minimal PSA throughout the follow-up period. PSA decline was calculated using the formula: (PSA at timepoint minus PSA at baseline)/PSA at baseline. The achievement of any PSA decline greater than 10% upon the first PSA measurement was defined as ‘any PSA decline’. PSA declines of 20% or 50% were calculated at nadir. Clinical benefit was subjectively assessed by the treating physician as an improvement in performance status, decline in pain, decline in narcotic consumption, or improvement in any cancer-related symptom. As the treatment was self-funded, patients were not automatically offered additional treatments beyond the first cycle. The decision whether to continue treatment was based on the subjective clinical benefit, the PSA decline, toxicity, the recovery of the blood counts, and on the patient’s wish.

### 2.4. Data Analysis

The primary objective of this study was to identify clinical, laboratory, or radiological variables associated with PSA response to [177Lu]-PSMA-617 treatment. Following the institutional review board’s ethical approval (the Sheba medical center (SMC) ethics committee, 6658-19-SMC), clinical data and lab results were extracted from the electronic medical record. When an exact date of an event could not be extracted, the event was determined to occur on the 15th of the month if known, or the 15th of July if only the year was known. The date of mCRPC was determined as the first PSA rise following the PSA nadir achieved on ADT, as long as a second PSA increase subsequently occurred. 

We calculated both the achievement of ‘any PSA decline’ on first measurement and the achievement of a 20% and 50% PSA decline at nadir following [177Lu]-PSMA-617 treatment. We analyzed associations between these response parameters and overall survival, which was calculated using the Kaplan–Meier method. Subsequently, potential variables associated with ‘PSA decline above 20%’ were analyzed by univariate analysis, using either logistic regression for continuous variables or χ^2^/Fisher’s exact test (depending on cell frequency) for categorical variables. Multivariable analysis was carried out using logistic regression on all variables with a *p*-value of <0.1 on univariate analysis or based on clinical importance. Continuous variables found to be statistically significant were then used to define a categorical score, using binary recursive partitioning to obtain the optimal cut-offs. All tests were two-tailed, with a probability of <0.05 considered statistically significant. Statistical analyses were performed using version 9.4 of the SAS System for Windows (Copyright © 2002-2012 SAS Institute, Inc., Cary, NC, USA), and the open source statistical software R version 3.6.3 (R Core Team, R Foundation for Statistical Computing, Vienna, Austria, 2020).

## 3. Results

Between February 2017 and July 2019, 52 men received at least one course of [177Lu]-PSMA-617 at Sheba Medical Center. The median age at which prostate cancer was diagnosed was 66.5 years (range 44.9–88.8), the median time from diagnosis to CRPC was 2.5 years (range 0.7–22.4 years), and the median time from CRPC to first [177Lu]-PSMA-617 treatment was 2.0 years (range 0.3–6.2). The median age at first [177Lu]-PSMA-617 treatment was 74.4 (range 56.6–91.9), and the median number of previous treatment lines for mCRPC was 4 (range 0–7); two patients (4%) received [177Lu]-PSMA-617 as either their first or second line, 8 (15%) received it as a third line, 16 (31%) received it as a fourth line, and 26 (50%) received it as their fifth line or beyond. The majority of men (84%) had received at least one line of previous chemotherapy, the majority of men (86%) had received at least one line of next generation hormonal agent (enzalutamide or abiraterone), and almost half (25 patients; 48%) had received prior treatment with radium-223. Most of the men (79%) had a compromised ECOG PS of at least 2, with 50% having an ECOG PS of 3 or 4. Twenty patients (38.5%) had visceral disease and almost half (*n* = 24, 46.2%) had metastasis to both bones and lymph nodes (Table 1).

Twenty nine patients (56%) received one treatment with [177Lu]-PSMA-617 at an average dose of 6.14 GBq (range 4.57–7.53 GBq), 12 patients (23%) received two treatments at an average dose of 6.28 GBq (range 5.71–7.25 GBq), 9 (17%) received three treatments at an average dose of 6.37 GBq (range 5.83–7.37GBq), and 2 (4%) patients received four [177Lu]-PSMA-617 treatments (at an average dose of 5.92 GBq). The timing of subsequent treatments beyond the first varied, with a median of 2.1 months (range 1.3–8.4) between the first and second cycles, and 3.7 months (range 1.9–5.7) between the second and third cycles. Median follow-up time was 3.6 months since the last treatment (range 0.2–24.6 months), and data cut-off was in mid-September 2019. Of all 52 treated patients, 44 (85%) had at least one documented PSA measurement post treatment. Six of the remaining patients had clinical deterioration or died prior to the PSA measurement, two more were lost during the follow-up period (most probably as a result of death), and all eight were considered non-responders.

**Figure 1:**
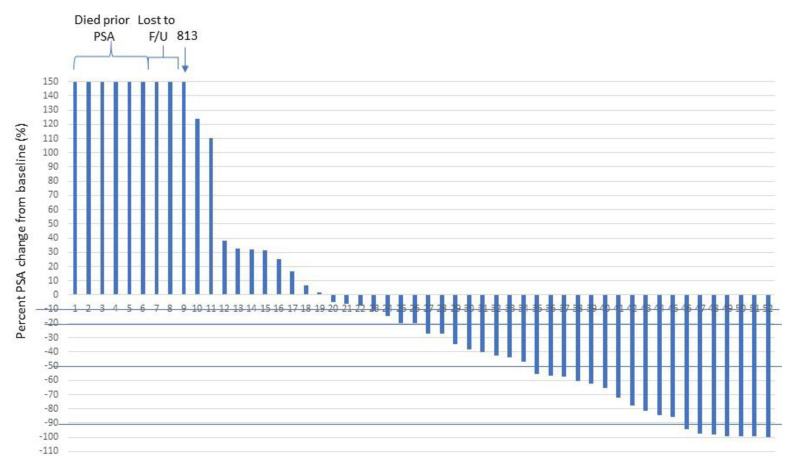
Percent change in PSA at nadir relative to baseline PSA (prior to first treatment).

A subjective clinical benefit, based on the clinical evaluation by the treating oncologist, was observed in 28 patients (52%). The ‘first PSA’ was measured following a median of 1.2 months from the start of treatment (range 0.4–3.7 months). PSA nadir was achieved within a median of 1.4 months (range 0.4–13.4). A maximal PSA decline of at least 20% was observed in 26 patients (50%), a maximal PSA decline of at least 50% was observed in 18 patients (35%), and a maximal PSA decline of at least 90% was observed in 7 patients (16%). Thirty patients (58%) had ‘any PSA decline’ (PSA decline greater than 10%) upon their first PSA assessment (Table 2). The maximal extent of PSA decline for all 52 patients is given in Figure 1.

**Figure 2:**
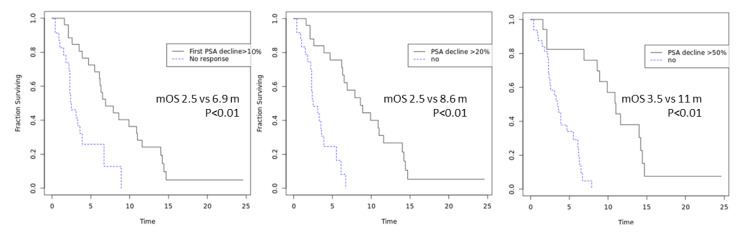
Overall survival in patients according to extent of PSA decline.

A maximal PSA decline of above 50% was significantly associated with OS, with a median survival time of 11 months vs 3.5 months for patients without such a PSA decline (*p* < 0.01). Similarly, a maximal PSA decline of above 20% at nadir was significantly associated with OS, with a median survival of 8.6 months vs 2.5 months in patients without such a PSA decline (*p* < 0.01). Interestingly, ‘any PSA decline’ upon first PSA measurement was also significantly associated with OS, with those achieving it having a median OS of 6.9 months vs 2.5 months in those who did not (*p* < 0.01; Figure 2).

**Figure 3:**
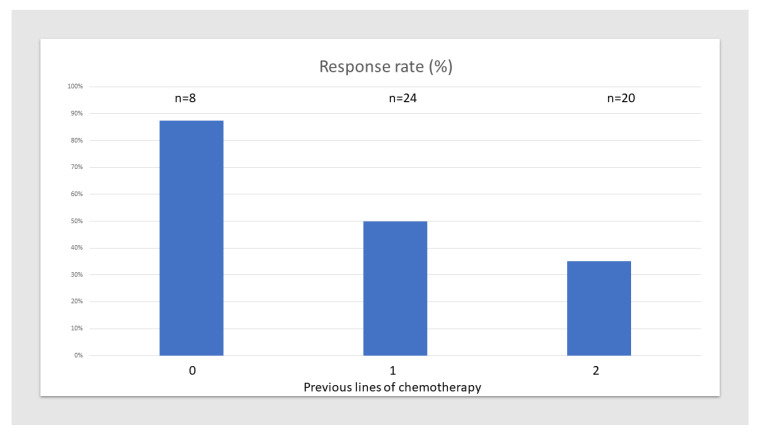
Response rate according to number of previous chemotherapy lines.

Since a PSA decline of at least 20% was seen in half of the patients, its association with clinical and laboratory variables was further studied using univariate analysis. The Gleason score, ECOG performance status (PS), metastatic sites, neutrophil–lymphocyte ratio (NLR), baseline PSA, lactate dehydrogenase (LDH), albumin, platelets, white blood cells (WBC), disease kinetics (time from diagnosis to CRPC, time from CRPC to treatment, or time from diagnosis to treatment), and treatment dose were not significantly associated with ≥20% PSA decline. Neither the number of total prior treatment lines for CRPC nor prior treatment with radium-223 were associated with it either, but there was a significant negative association between the number of previous chemotherapy lines (ranging from 0–2) and a PSA decline of 20% or more (*p* = 0.043; Figure 3).

Older age, higher hemoglobin, and lower baseline alkaline phosphatase were each positively associated with a PSA decline >20% in univariate analysis (Table 3). 

Optimal cut-offs for each of these variables were found to be age above 77 years, hemoglobin equal or above 9.2 gr/dL, and alkaline phosphatase below 260 IU/L. Multivariable analysis for these three dichotomous variables showed that older age and higher hemoglobin were associated with a PSA response above 20% (with corresponding p values of 0.03 and 0.006, respectively) but alkaline phosphatase (*p* = 0.09) was not.

Lastly, we generated a score combining two clinical parameters: age > 77 and hemoglobin ≥ 9.2 gr/dl, which ranged from 0–2. Of 47 patients who had a documented baseline hemoglobin, 14 (30%), 22 (47%), and 11 (23%) patients had calculated scores of 0, 1, and 2, respectively. This score was significantly associated with a PSA decline >20%: a 7.1%, a 63.6%, and a 81.8% response rate for the three score groups, respectively (*p* = 0.0003; Table 4).

## 4. Discussion

[177Lu]-PSMA-617 is a novel treatment modality for prostate cancer, utilizing the tumor’s expression of PSMA as a target to allow for the administration of mixed beta–gamma radiation directly to the site of active metastases. This treatment is only given in several countries, either in an off-trial or on-trial setting, as it is not a standard of care anywhere to date. Here, we present our initial clinical experience with 52 patients treated with [177Lu]-PSMA-617 after exhaustion of all lines of therapy for mCRPC for which they were deemed fit. Indeed, the patients in this cohort were heavily pre-treated, with a median number of 4 previous lines of therapy, and with generally compromised or extremely compromised performance status.

A maximal PSA decline of at least 50% was seen in 35% of the patients; this is numerically lower than the rate in the published phase II trial [5] and may very well be explained by the differences in the patient characteristics, namely, a more advanced line meant worse baseline laboratory values (lower hemoglobin and higher alkaline phosphate and LDH) and the compromised performance status of the patients in our cohort. In addition, the lower rate of PSA decline in our cohort may have also stemmed from the fact that since the treatment was self-funded and given as a ‘last line’, the timing of subsequent treatments was not strict, and treatments may well have occurred following PSA progression and not every 6 weeks as in the phase II trial [5]. The response rate seen in our cohort resembles (although is slightly lower than) the calculated response rate of 43% across twelve studies including 669 patients [6]. A similar rate of PSA decline above 50% was also described Heck et al. [3].

About half of the patients were thought to have had a clinical benefit from the treatment, similar to the results from the phase II trial [5] and similar to other published retrospective studies and meta-analyses [6,7].

Maximal PSA declines of >20% or >50% were both associated with OS. A similar observation was also seen in a post hoc analysis in the phase II trial. Interestingly, a PSA decline of at least 10% at first assessment following the first treatment was also associated with OS. This is in agreement with the observation by Gafita et al., showing that an early decrease of PSA by 6 weeks is associated with survival [8]. This may suggest that the achievement of any PSA decline (as opposed to continued increase or stabilization post-treatment) may serve as a surrogate for benefit from treatment. Clearly, this must be studied prospectively. If such an association will be evident in a prospective trial, then it may have implications for patient selection for subsequent cycles, thus allowing us to withhold treatment from those patients not likely to benefit and continuing only in those cases with a high likelihood of benefit. This will also have implications for treatment cost and reimbursement.

Four clinical variables were found to be associated with a PSA decline of above 20% in univariate analysis: age, baseline hemoglobin, baseline alkaline phosphatase, and the number of prior chemotherapy lines, of which two, age and baseline hemoglobin, were found to be statistically significant in multivariable analysis. This enabled the generation of a clinical score of three tiers that was significantly associated with a decline in PSA of above 20%. Clearly, this clinical score needs to be prospectively validated. Previous work by Ferdinandus et al. also found that higher age and higher hemoglobin were associated with PSA response to [177Lu]-PSMA-617 [9], but these were not found to be significant in their multivariable analysis. The difference between their results and ours may perhaps stem from their smaller cohort of 40 patients. Also, similar to our observations, response in their cohort was not associated with the number of previous lines of therapy [9]. A recent work from the same group showed that additional disease-related parameters, such as the FDG-avid tumor volume, the intensity of the PSMA uptake, and the bone-scan index are also associated with overall survival [10].

The negative association between the number of previous chemotherapy lines and PSA response may suggest cross-resistance between chemotherapy and [177Lu]-PSMA-617. This was also suggested in a retrospective analysis of taxane-pre-treated and taxane-naïve mCRPC patients [11], and in a retrospective analysis by Kessel et al. [12]. However, the total number of previous lines of any sort (chemo, hormonal, or radio-nuclide) was not associated with response, further corroborating the efficacy of [177Lu]-PSMA-617 in advanced treatment lines. This is consistent with the lack of association between PSA response and the duration of the disease (measured as either time from diagnosis to CRPC, time from CRPC to treatment, or time from diagnosis to treatment). Further research is needed in order to find the biological/pathophysiological determinants of response to [177Lu]-PSMA-617.

Our work has several clear limitations; first, the cohort of patients is small and derived from a single center. Indeed, it is difficult to obtain larger cohorts from many centers, as this treatment is not standard and only given in selected centers. The largest prospective trial to date included 30 treated patients (of 43 screened) in a single center [5], and the published retrospective cohorts so far include any number of patients from a dozen or two to one hundred. Having said that, it is reassuring to see good consistency in the clinical results across the different retrospective cohorts in different continents and across different practices [6,7,8,9,10,11,12]. Second, eight patients did not have any post-treatment PSA, of which six died prior subsequent PSA measurement and two were lost to follow-up. All of these were considered to have progressed, but this may have still introduced a bias. Lastly, the administration of [177Lu]-PSMA-617 was not always given every 6–8 weeks as per protocol; rather, it was based on additional paramedical parameters such as cost and patient wish. The implications of this difference in dosing intensity are unknown, yet they may also suggest that less frequent administration is feasible in this end-stage setting, with the achievement of significant palliative benefits, nonetheless, and at a lower cost.

## 5. Conclusions

Our work adds to the body of accumulating evidence showing that [177Lu]-PSMA-617 is active in advanced lines in mCRPC and suggests that a PSA decline (either immediate, of any magnitude of at least 10%, or of at least 20% at PSA nadir) is associated with survival benefit. Our analysis also points to two clinical parameters—older age and higher hemoglobin—associated with PSA response to be further studied prospectively.

## Figures and Tables

**Table 1:** Patient characteristics.

Characteristics	Distribution	Median	Range
Age at diagnosis (Dx; years)		66.5	44.9–88.8
Gleason score at Dx	6–4% 7–20% 8–13% 9–37% 10–6% NA–21%	9	6–10
Time from Dx to CRPC (years)		2.5	0.7–22.4
Time from CRPC to first Lutetium treatment (years)		2	0.3–6.2
Age at first Lutetium-177-PSMA treatment (years)		74.4	56.6–91.9
Number of treatment lines for mCRPC	0–2% 1–2% 2–15% 3–31% 4–29% 5–21% 7–2%	4	0–7
Number of previous chemo lines	0–15% 1–46% 2–38%	1	0–2
Hemoglobin (gr/dL)		10.1	6.2–13.7
WBC (K/microL)		5.8	1.6–19.0
PLTs (K/microL)		212	12–440
PSA (micg/L)		131	0.4–6300
Lactate Dehydrogenase (LDH; IU/L)		373	123–7280
Alkaline phosphatase (IU/L)		186	48–1188
		*n*	%
Metastatic sites	Bone or LN only	8	15.4
	Bone+ Lymph nodes	24	46.2
	Visceral (+/- bone/LNs)	20	38.5
ECOG PS	0–1	11	21.2
	2	15	28.8
	3–4	26	50

**Table 2:** PSA response characteristics.

	Responders *n* (%)	95% CI (%)
Any PSA decline at first PSA (≥10%)	30 (58%)	47–69
Decline >20% at nadir	26 (50%)	38–62
Decline >50% at nadir	18 (35%)	25–45
Decline >90% at nadir	7 (16%)	9–23

**Table 3:** Clinical factors associated with PSA decline >20% by univariate analysis.

Parameter	*p*-Value	OR	95% CI	
			Lower	Upper
Age	0.0515	1.071	1.000	1.147
Hemoglobin	0.0240	1.533	1.058	2.220
Alkaline Phosphatase	0.0283	0.994	0.989	0.999

**Table 4:** Response rate according to score based on age and hemoglobin.

Score	Description	*N* (%)	Responders (*N*)	Response Rate (%)	95% CI (%)	*p* Value
0	Age < 77 and Hemoglobin <9.2 gr/dL	14 (30)	1	7.1	0–20.6	0.0003
1	Age > 77 and Hemoglobin <9.2 gr/dL, or Age < 77 and Hemoglobin ≥9.2 gr/dL	22 (47)	14	63.6	43.5–83.7	
2	Age > 77 and Hemoglobin ≥9.2 gr/dL	11 (23)	9	81.8	59–1.0	
